# Irisin Ameliorates Oxidative Stress-Induced Injury in Pancreatic Beta-Cells by Inhibiting Txnip and Inducing Stat3-Trx2 Pathway Activation

**DOI:** 10.1155/2022/4674215

**Published:** 2022-09-06

**Authors:** Chongxiao Liu, Jianhua Zhou, Yanhong Xu, Sa Gong, Yi Zhu, Hongli Zhang, Yan Dong, Bingxia Zhao, Xiaohua Li

**Affiliations:** ^1^Department of Endocrinology, Seventh People's Hospital of Shanghai University of Traditional Chinese Medicine, Shanghai 200137, China; ^2^Xinhua Hospital, Shanghai Jiao Tong University School of Medicine, Shanghai 200092, China; ^3^Shanghai Institute for Pediatric Research, Shanghai 200092, China; ^4^Center of Minimally Invasive Treatment for Tumor, Department of Medical Ultrasound, Shanghai Tenth People's Hospital, School of Medicine, Tongji University, Shanghai 200072, China

## Abstract

Lipotoxicity can lead to beta-cell dysfunction and apoptosis because it induces oxidative stress. Recent studies have found that Irisin prevents pancreatic beta-cell dysfunction induced by palmitic acid (PA). However, an association between the protection against oxidative stress conferred by Irisin and beta-cell dysfunction has not been fully elucidated. In this study, we observed that Irisin treatment prevented INS-1 cell apoptosis induced by PA treatment and preserved the insulin-secreting function of INS-1 cells in vitro. These effects probably resulted from the Irisin-induced decrease in intracellular ROS levels triggered by PA treatment. In addition, PA treatment induced oxidative stress partially by inhibiting the activation of thioredoxin 2 (Trx2) through its increase of thioredoxin-interacting protein (Txnip) expression. However, Irisin administration blocked the increase in Txnip expression, which reversed the PA-induced inactivation of Trx2. Irisin also increased the nuclear translocation of Stat3, and the inhibition of Stat3 by siRNAs blocked Irisin-induced Trx2 expression, indicating that both Txnip and Stat3 are involved in Irisin-induced activation of Trx2. Furthermore, blockade of Stat3 by siRNAs led to the decreased gene expression of *MafA* and *Ins* and to cessation of glucose-induced insulin secretion that had been enhanced by Irisin. In vivo, HFD treatment led to reduced glucose tolerance and an increase in the level of the oxidative marker malondialdehyde (MDA) compared to that in the control group. However, these effects were ameliorated by Irisin injection due to the inhibition of beta-cell apoptosis and the activation of Trx2, probably through Txnip inhibition and Stat3 activation. In conclusion, our results reveal a possible mechanism for Irisin-induced beta-cell protection, which is mediated through Txnip inhibition and activation of the Stat3-Trx2 pathway.

## 1. Introduction

Type 2 diabetes is characterized by progressive pancreatic beta-cell dysfunction and peripheral insulin resistance. Obesity, characterized by elevated levels of circulating free fatty acids (FFAs) and dysregulation of lipid metabolism, is associated with the pathogenesis of type 2 diabetes [1]. As previously reported, chronic exposure to excess lipids causes severe damage to the insulin-secreting function of pancreatic beta-cells and increases beta-cell death, an effect known as lipotoxicity [2]. Oxidative stress is an important mechanism of lipotoxicity in beta-cell dysfunction. High levels of FFAs significantly increase intracellular ROS levels, further disrupting insulin secretion and causing beta-cell apoptosis, ultimately leading to the development of diabetes [2, 3]. However, to date, few antidiabetic drugs have conferred oxidative injury induced by lipotoxicity protection on beta-cells. Therefore, it is of great importance to find a way to prevent lipotoxicity-induced beta-cell damage and thus delay the progression of diabetes.

Several clinical studies have found that the level of Irisin was significantly lower in type 2 diabetic patients than in healthy control individuals and was negatively correlated with blood glucose, lipids, and visceral fat deposition [4, 5]. Irisin, a newly discovered myokine, is a secretable polypeptide fragment formed by the hydrolysis of fibronectin type III domain-containing protein 5 (FNDC5) [6]. Exercise promotes the expression of PPAR-*γ* coactivator-l*α* (PGC-1*α*) in skeletal muscle cells, which upregulates the expression of FNDC5. After excision of the N-terminal signal peptide, FNDC5 is hydrolyzed to form a polypeptide fragment of approximately 110 amino acids, known as Irisin [7]. A high level of Irisin in mice has also been found to increase the expression of uncoupling protein 1 (UCP1) in adipocytes, promote the conversion of white fat to brown fat, increase energy expenditure, reduce obesity caused by a high-fat diet, and enhance glucose tolerance [7]. In addition to its positive effects on energy metabolism, Irisin has been discovered to confer protection against oxidative stress. Irisin ameliorated increased ROS production in endothelial cells caused by glucolipotoxicity and further enhanced endothelial function through activation of the PKC-*β*/NADPH oxidase and NF-*κ*B/iNOS pathways [8]. Another study found that Irisin attenuated oxidative stress injury by activating superoxide dismutase 1 (SOD1) in cardiomyocytes and repaired mitochondrial function destroyed by myocardial ischemia reperfusion [9]. These studies suggested that Irisin is involved in the regulation of intracellular redox homeostasis and might play an important role in the resistance to cellular mitochondrial damage. Recent studies also reported that Irisin exerted a protective effect on glucolipotoxicity-induced beta-cell dysfunction [10, 11]; however, there are few reports related to the improvement of oxidative stress damage in beta-cells by Irisin or the underlying mechanisms.

The thioredoxin system, comprising thioredoxin (Trx), thioredoxin reductase (TrxR), and peroxiredoxin (Prx), serves as an important regulator of intracellular ROS reduction, which involves the cytosolic Trx1 system and mitochondrial Trx2 system. Trx2, encoded by the nuclear gene *Txn2*, is part of the mitochondrial Trx2 system and plays a significant role in the regulation of intracellular redox homeostasis [12]. Moreover, Trx2 plays an antiapoptotic role by binding with apoptosis signal-regulating kinase 1 (ASK1) [13]. The activity of Trx2 can be inhibited by thioredoxin-interacting protein (Txnip), a natural inhibitor of Trx [14], and increased expression of Txnip has recently been found to contribute to high-glucose-induced beta-cell injury [15]. Our previous study confirmed that Trx2 inhibition was involved in high-glucose-induced oxidative injury in INS-1 cells, in which it damaged normal mitochondrial function [16]. However, we have no clue about the role played by Trx2 in lipotoxicity-induced oxidative stress in beta-cells. Whether Trx2 is a contributor to PA-induced oxidative damage of beta-cells and a mediator of Irisin-induced antioxidative effects needs to be elucidated.

Signal transducer and activator of transcription 3 (Stat3) belongs to the superfamily of signal transducer and activator of transcription proteins. Recently, Stat3 has been proven to protect cell function through regulation of mitochondrial oxidative respiratory chain complex I in postischemic cardiomyocytes [17]. Stat3 inhibition increased the ROS contents in mitochondria and accelerated the apoptosis of pancreatic cancer cells [18]. A recent study discovered that Stat3 was involved in maintaining normal mitochondrial function in islet beta-cells and suggested that its deficiency might induce glucose intolerance in obesity [19]. Stat3 participates in the regulation of ROS production and mitochondrial function, but the underlying mechanism is unclear. Irisin has also been reported to be involved in the activation of Stat3 [20], and our preliminary experiments revealed that Irisin treatment increased the expression of mitochondrial Trx2 in pancreatic beta-cells. Therefore, we doubt that Stat3 activation participates in the activation of mitochondrial Trx2 induced by the Irisin treatment of beta-cells.

In this study, we hypothesized that the protective effects of Irisin in pancreatic beta-cells might be attributed to Trx2 activation and inhibition of oxidative stress. We thus assessed the potential for Irisin to regulate Trx2 activation through the inhibition of Txnip expression and the activation of the Stat3 pathway.

## 2. Materials and Methods

### 2.1. Animals and Treatment

Male C57BL/6J mice (6-8 weeks) were purchased from the Shanghai SLAC Laboratory Animal Company (Shanghai, China). All procedures for animal experiments were approved by the University Animal Use Committee (No. XHEC-NSFC-2018-259) and performed in accordance with its experimental animal care guidelines. Animals were housed in a specific-pathogen-free (SPF) environment with a 12 h light-dark cycle and access to food and water ad libitum. After acclimatization for one week, all mice (*n* = 30) were randomly assigned to a group with access to a standard diet (*n* = 10, control group) or a high-fat diet (*n* = 20, HFD group) in which 60% of the total kilocalories are obtained from saturated fat (Research Diets, New Brunswick, NJ, D12492) for 12 weeks. At the end of the intake period, 10 mice in the HFD group were intraperitoneally (IP) administered Irisin dissolved in saline at 500 *μ*g/kg (time points: days 1, 3, 5, 7, 9, 11, 13, 15, 17, 19, 21, 23, 25, and 28 at 10:00 a.m.), and the other mice in the HDF group were administered the corresponding amount of saline solution. All mice were maintained on their respective diets for four weeks. After 4 weeks of diet administration, intraperitoneal glucose tolerance tests (IPGTTs) were preformed, and 3 days later, these mice were euthanized through an intraperitoneal injection of 2% pentobarbital sodium after overnight fasting. Blood samples were collected, and the pancreas was resected and frozen in liquid nitrogen for further measurement.

### 2.2. Intraperitoneal Glucose Tolerance Tests (IPGTTs)

IPGTTs were performed after overnight fasting. Animals were intraperitoneally injected with glucose at a dose of 2 g/kg of body weight. Blood samples were taken from the tail 0, 15, 30, 60, and 120 min after injection, and the glucose levels were analyzed with a OneTouch Ultra system (Roche, Germany). The area under the curve (AUC) of the IPGTT data was calculated following the formula AUC (mmol/L/h) = (BG0 + BG15) × 15/120 + (BG15 + BG30) × 15/120 + (BG30 + BG60) × 15/60 + (BG60 + BG120) × 30/60.

### 2.3. Cell Culture and Treatment

INS-1 cells, a rat pancreatic beta-cell line donated by Shanghai Institute of Endocrine and Metabolic Diseases, were cultured in RPMI 1640 as previously reported [21]. PA powder was dissolved in 0.1 M sodium hydroxide to prepare a stock solution (100 mM), and fatty acid-free BSA (Roche, USA) was diluted in double-distilled water to prepare 5% BSA (wt/vol). A 5 mM PA/5% BSA solution was prepared by adding the appropriate amount of PA to 5% BSA and then diluting the mixture in RPMI 1640 to reach the desired final concentrations after cooling to room temperature. The control group was given BSA solution at the corresponding concentration. Irisin (Phoenix Pharmaceuticals, USA) was dissolved in double-distilled water to prepare a stock solution (20 mM) and diluted in RPMI 1640 to the desired final concentrations. In some experiments, cells were pretreated with an anti-Irisin neutral antibody (Irisin NA, Phoenix Pharmaceuticals, USA) for 30 min.

### 2.4. Small Interfering RNA (siRNA) Transfection

Stat3 siRNAs were used to reduce Stat3 expression. Briefly, a transfection mixture containing three specific Stat3 siRNAs (siRNA1, siRNA2, and siRNA3) and siRNA transfection reagent (Invitrogen, USA) was incubated for 20 min at room temperature, and the cells were then incubated with this mixture for 6 h at 37°C. The control containing nontargeting scramble siRNA was incubated in parallel. The following siRNA sequences (5′ to 3′) were synthesized by GeneChem (Shanghai, China):

siRNA1, GAAGCCAATGGAAATTGCCCGGATT

siRNA2, GGAGGAGAGGATCGTGGATCTGTT

siRNA3, GGAGGAGGCATTCGGAAAGTATT for *Stat3*

### 2.5. Cell Viability Determination

A cell viability assay (CCK-8, Servicebio, China) was preformed to measure the protective effect exerted by Irisin on INS-1 cells. According to the manufacturer's instructions, cells were seeded in a 96-well plate at a density of 10^4^ cells per well, and 10 *μ*L of CCK-8 solution was added to each well after treatment. The cells were then incubated for 1 hr at 37°C, and the absorbance was measured at a wavelength of 450 nm with a microplate spectrophotometer (Biotek, USA). Cell viability is presented as the fold change in expression compared to the control. Each experiment was replicated at least three times.

### 2.6. ROS Detection

Both cytosolic and mitochondrial ROS were measured with 2′,7′-dichlorodihydrofluorescein-diacetate (DCFH-DA, at a wavelength of 525 nm, Sigma-Aldrich, USA) and MitoSOX (a probe of mitochondrial ROS, at a wavelength of 580 nm, Invitrogen, USA), respectively. After incubation with 10 *μ*M DCFH-DA for 30 min or 5 *μ*M MitoSOX for 15 min in a dark room, treated INS-1 cells were washed twice with RPMI 1640 containing no FBS and immediately analyzed to evaluate the levels of ROS with either a Synergy H4 Multi-Mode Microplate Reader (Biotek, USA) or fluorescence microscope (Leica, Germany).

### 2.7. Superoxide Anion Detection

Superoxide content in INS-1 cells was detected with a Superoxide Assay Kit (Beyotime Biotechnology, China) according to the manufacturer's instructions. The superoxide level was presented as the OD_450_ based on the measurements obtained with the microplate reader (Biotek).

### 2.8. Immunofluorescence Analysis

Insulin and glucagon contents in pancreatic islets were determined through an immunohistofluorescence analysis. Briefly, fresh pancreatic tissues were fixed in 10% paraformaldehyde and embedded prior to sectioning. All sections were incubated with secondary antibody (Jackson ImmunoResearch, USA) in the dark for 1 hr at room temperature and dyed with DAPI after staining with primary antibody (anti-insulin antibody (CST, USA) and anti-glucagon antibody (Abcam, USA)) at 4°C overnight.

The insulin content was measured, and Stat3 translocation in INS-1 cells was detected through an immunocytofluorescence analysis. INS-1 cells were seeded on circular slides in a 6-well plate. After the corresponding treatment, the cells were fixed in 4% paraformaldehyde for 15 min and blocked with 5% BSA for 30 min, followed by incubation with the corresponding primary antibody (anti-insulin antibody (CST, USA) and anti-Stat3 antibody (Abcam, USA)) at 4°C overnight, and the cells were then incubated with FITC-tagged secondary antibody (Jackson ImmunoResearch, USA) in the dark for 1 hr at room temperature and dyed with DAPI to capture the distribution of fluorescence under a fluorescence microscope (Olympus, Japan).

### 2.9. Glucose-Stimulated Insulin Secretion

Glucose-stimulated insulin secretion (GSIS) was performed as reported previously [16]. The medium was collected, and the insulin level was measured using an insulin ELISA kit (Roche Applied Science, USA) following the manufacturer's instructions.

### 2.10. Immunoblot Analysis

Total proteins in INS-1 cells and pancreatic tissues were extracted using radioimmunoprecipitation assay (RIPA) lysis buffer (Beyotime Biotechnology, China), and protein expression was determined by immunoblotting. The primary antibodies used were anti-Trx2, anti-Txnip, anti-Stat3, and anti-p-Stat3 (Abcam, USA); anti-Tubulin (CST, USA); and anti-*β*-actin (Sigma, USA) antibodies.

### 2.11. Thioredoxin 2 Activity Assay

Mitochondrial thioredoxin 2 activity was determined with an insulin disulfide reduction assay as reported previously [16]. Mitochondrial protein was extracted with a mitochondrial protein extraction kit (Beyotime Biotechnology, China), and Trx2 activity is presented as the OD_412_ fold change compared to the control on the basis of the absorption measurement obtained at a wavelength of 412 nm.

### 2.12. Quantitative Real-Time PCR (qRT-PCR) Analysis

Gene expression in INS-1 cells was detected by quantitative real-time PCR. Briefly, a total RNA sample was extracted, and cDNA was synthesized from 1 *μ*g of RNA with PrimeScript RT Master Mix (TaKaRa Biomedicals, Osaka, Japan). A qRT-PCR analysis was then performed with SYBR Premix Ex Taq (TaKaRa Biomedicals) following the manufacturer's instructions. The primers for rat (designed by Sangon Biotech, Shanghai, China) were synthesized following the sequences in Table 1.

### 2.13. Apoptosis Analysis

Beta-cell apoptosis of pancreatic islets was detected by terminal deoxynucleotidyl transferase-mediated dUTP nick-end labeling (TUNEL) staining following the instructions in an In Situ Cell Death Detection Kit (Roche Biochemicals, USA). The beta-cell nuclei were counterstained with DAPI and stained for insulin. The samples were visualized, and digital images were acquired by fluorescence microscopy.

The apoptosis of the INS-1 cells was measured with a Cell Death Detection ELISA Kit (Roche Biochemicals) following the manufacturer's protocol.

### 2.14. Estimation of Oxidative Stress Markers in Pancreatic Tissue

The particular reaction of ROS with lipids is generally known as “lipid peroxidation.” Malondialdehyde (MDA) is formed through lipid peroxidation and is widely accepted as a biomarker of oxidative stress [22]. Total proteins in the mouse pancreas of the three groups and INS-1 cells were extracted using RIPA lysis buffer (Beyotime Biotechnology, China) and adjusted to be the same concentration (1 mg/mL). The level of MDA was measured via a lipid peroxidation MDA assay kit (Beyotime) following the manufacturer's recommendations. In addition, the activity levels of superoxide dismutase (SOD) and glutathione peroxidase (GPx) in the pancreas were measured using GPx and SOD assay kits (Beyotime).

### 2.15. Statistical Analysis

Data are expressed as mean ± SEM, and an independent sample *t* test was performed for a comparison between two groups after normality was corroborated. For multigroup comparisons, one-way ANOVA tests were performed, and a least significant difference (LSD) test was performed for a comparison between the two groups. At least three independent experiments were conducted in our study. All statistical analyses were performed using SPSS statistical software (v19.0), and *P* values < 0.05 were considered to be statistically significant.

## 3. Results

### 3.1. Irisin Exerts Protective Effects against Apoptosis and Dysfunction in INS-1 Cells after PA Treatment

**Figure 1:**
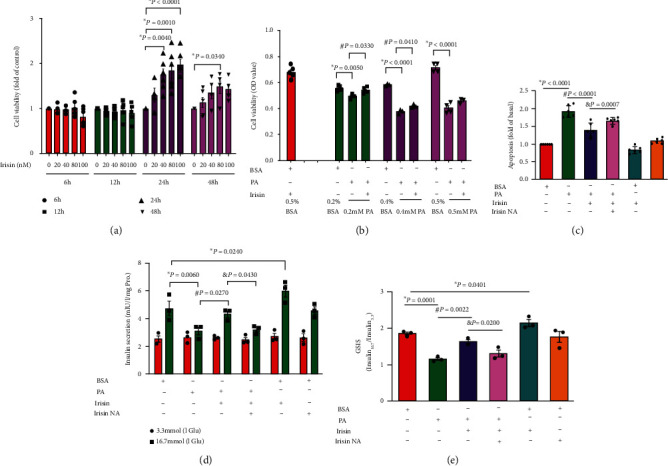
Irisin exerted protective effects on INS-1 cell apoptosis and dysfunction under PA treatment. (a) Cell viability analysis with Irisin treatment at different concentrations from 6 hrs to 48 hrs; the control at 0 nM (*n* ≥ 3 independent experiments). (b) Cell viability analysis with PA and Irisin cotreatment for 24 hrs, PA at 0.2 mM, 0.4 mM, and 0.5 mM; Irisin at 100 nM; corresponding BSA volumes were set as controls (*n* = 3 independent experiments). (c) INS-1 cell apoptosis with PA and Irisin cotreatment for 24 hrs, PA at 0.2 mM; Irisin at 100 nM; Irisin NA at 100 nM; BSA was the control (*n* = 6 independent experiments). (d) Insulin secretion after glucose stimulation was measured by ELISA. (e) The secretory function of INS-1 cells is presented as the ratio of insulin, that is, secretion after 16.7 mM glucose treatment to that secreted after 3.3 mM glucose treatment (*n* = 3 independent experiments). The data are expressed as means ± SE; ∗*P* < 0.05*vs.* the control; ^#^*P* < 0.05 vs. the PA treatment group; ^&^*P* < 0.05 vs. the PA and Irisin cotreatment group. BSA: bovine serum albumin; PA: palmitic acid; Irisin NA: anti-Irisin-neutralizing antibody.

INS-1 cells were treated with different concentrations of recombinant Irisin at different time points, and a significant time-dependent and concentration-dependent increase in cell viability was observed (Figure 1(a)). After 100 nM Irisin treatment for 24 hrs, cell viability was increased more than twofold compared to that in the control group. Furthermore, PA treatment significantly reduced the viability of INS-1 cells and increased their apoptosis rate (Figures 1(b) and 1(c)), but the apoptosis of INS-1 cells was markedly decreased in the PA and Irisin cotreatment group compared with the PA single-treatment group. However, Irisin NA treatment significantly blocked this decrease in cell apoptosis, further proving that Irisin plays a protective role against PA-induced cell apoptosis. In addition, Irisin treatment increased insulin secretion by INS-1 cells in response to 16.7 mM glucose stimulation, which was blocked by PA administration and similarly impeded by Irisin NA cotreatment (Figures 1(d) and 1(e)).

### 3.2. Irisin Decreases ROS Levels Induced by PA Treatment in INS-1 Cells

**Figure 2:**
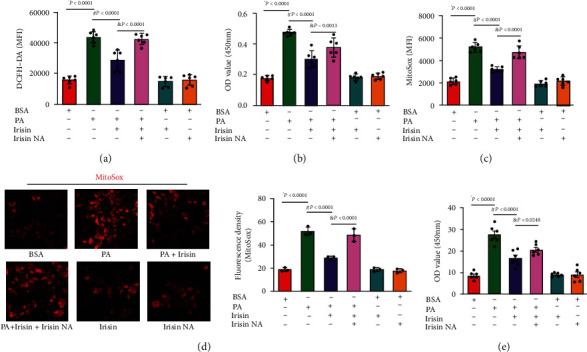
Irisin protected INS-1 cells from oxidative stress. PA at 0.2 mM; Irisin at 100 nM; Irisin NA at 100 nM; BSA set as the control. (a) Levels of cytosolic ROS were detected with a DCFH-DA probe. The DCF green signal was captured, and the MFI (mean fluorescence intensity) was calculated and is shown in histogram to quantify intracellular ROS (*n* = 6 independent experiments). (b) Superoxide content in INS-1 cells with PA and Irisin cotreatment is presented as the OD value at 450 nm and is shown in bar chart (*n* = 6 independent experiments). (c) Mitochondrial ROS levels were detected with the MitoSOX probe, and the MFI calculated was calculated and is shown in histogram to quantify the levels of mitochondrial ROS (*n* = 6 independent experiments). (d) Mitochondrial ROS tagged by MitoSOX were captured by fluorescence microscope (×100), and the fluorescence intensity is shown in histogram (*n* = 3 independent experiments). (e) MDA content in INS-1 cells in different groups (*n* = 6 independent experiments). The data are expressed as mean ± SE; ∗*P* < 0.05 vs. the control; ^#^*P* < 0.05 vs. PA treatment group; ^&^*P* < 0.05 vs. PA and Irisin cotreatment group. BSA: bovine serum albumin; PA: palmitic acid; Irisin NA: Irisin-neutralizing antibody.

Oxidative stress plays an important role in PA-induced beta-cell dysfunction. In our study, we detected an increase in the levels of intracellular ROS and superoxide after PA treatment for 24 hrs (Figures 2(a) and 2(b)), and this increase was blocked by Irisin exposure in INS-1 cells. Cotreatment of INS-1 cells with Irisin NA markedly retarded Irisin-induced ROS decline. Furthermore, a significant decrease in mitochondrial ROS levels was detected by the MitoSOX probe in the PA and Irisin cotreatment group compared to the PA group, as shown in Figures 2(c) and 2(d), and this decrease was also blocked by Irisin NA cotreatment. In addition, PA treatment triggered MDA formation, indicating overactivation of lipid peroxidation, but Irisin cotreatment prevented this increase in MDA in INS-1 cells (Figure 2(e)).

### 3.3. Irisin Protects INS-1 Cells from Oxidative Stress through Trx2 Activation

**Figure 3:**
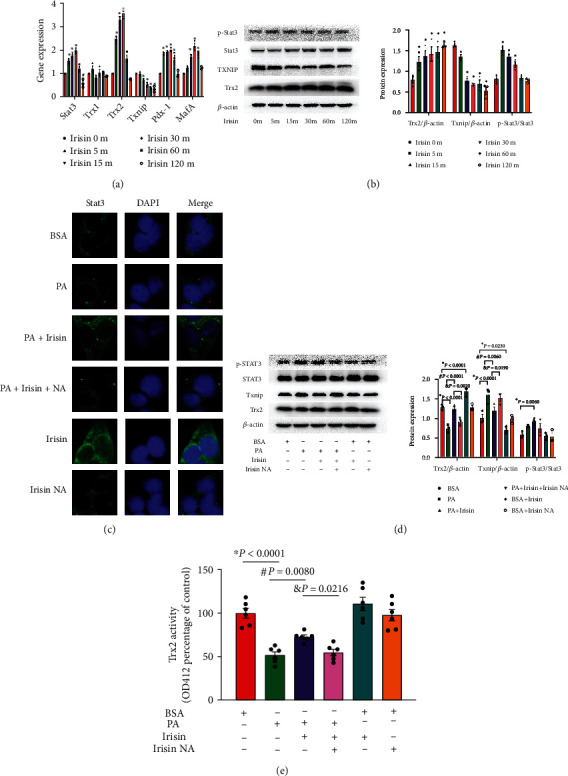
Trx2 was activated by Irisin through Stat3 nuclear translocation and Txnip inhibition of PA at 0.2 mM, Irisin at 100 nM, and Irisin NA at 100 nM; BSA was used as the control. (a) Gene expression of *Trx1*, *Trx2*, *Txnip*, *MafA*, *Pdx-1*, and *Stat3* was measured by qPCR after Irisin treatment of INS-1 cells. *β*-Actin was used as an internal standard (*n* = 4 independent experiments). (b) Protein expression of Trx2, Txnip, p-Stat3, and Stat3 was measured by immunoblotting after Irisin treatment in INS-1 cells and is presented in a histogram (*n* = 3 independent experiments). *β*-Actin was the internal standard. (c) Stat3 nuclear translocation in INS-1 cells was detected by immunofluorescence analysis and captured by fluorescence microscopy (×100) (*n* = 3 independent experiments). (d) Protein expression of Trx2, Txnip, p-Stat3, and Stat3 was detected after PA and Irisin cotreatment and is shown in the histogram (*n* = 3 independent experiments). (e) Mitochondria were separated and detected with an insulin disulfide reduction assay to determine Trx2 activity (*n* = 6 independent experiments). The data are expressed as the mean ± SE; ∗*P* < 0.05 vs. the control; ^#^*P* < 0.05 vs. the PA treatment group; ^&^*P* < 0.05 vs. the PA and Irisin cotreatment group. BSA: bovine serum albumin; PA: palmitic acid; Irisin NA: anti-Irisin-neutralizing antibody.

To further explore the underlying mechanism of Irisin on the inhibition of oxidative stress in INS-1 cells, we found that Irisin administration dramatically increased the expression of Trx2 but not Trx1, suggesting that mitochondrial Trx2 activation might be critical to the ROS blockage caused by Irisin (Figure 3(a)). Both Pdx-1 and MafA play crucial roles in insulin biosynthesis and secretion [23, 24]. Increased expression levels of Pdx-1 and MafA were also measured in our study, and the results indicated that Irisin enhanced insulin biosynthesis. We further discovered that Irisin increased Stat3 phosphorylation and inhibited Txnip expression (Figures 3(b) and 3(c)). PA treatment also prevented Trx2 expression, probably due to increased expression of Txnip but not Stat3 inactivation, as shown in Figure 3(d). Irisin blocked the Trx2 inhibition induced by PA treatment through Stat3 nuclear translocation and Txnip inhibition, but these outcomes were reversed by Irisin NA cotreatment (Figures 3(c) and 3(d)). Trx2 activity in isolated mitochondria was inhibited by PA but was recovered with Irisin cotreatment, as shown in Figure 3(e). Irisin NA administration retarded the Irisin-induced increase in Trx2 activity, suggesting that Irisin-induced ROS inhibition might result from Trx2 activation by inhibiting Txnip and promoting Stat3 nuclear translocation.

### 3.4. Trx2 Is Activated by Irisin through Stat3 Nuclear Translocation and Txnip Inhibition

**Figure 4:**
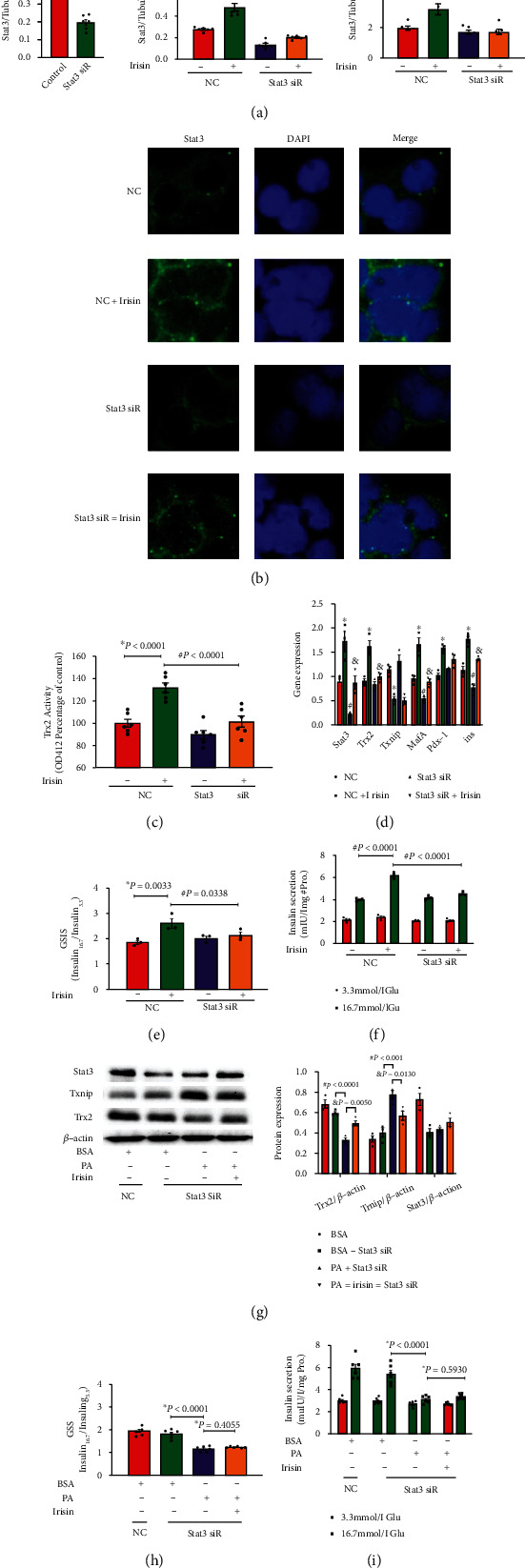
Stat3 inhibition by Stat3 siRNAs blocked Irisin-induced Trx2 activation and insulin secretion in INS-1 cells. Irisin at 100 nM with NC set as the control. (a) Protein expression of Trx2 and Stat3 in INS-1 cells with Stat3 siRNAs was measured by immunoblotting and is shown in histograms (*n* = 4 independent experiments). (b) Trx2 activity was detected by insulin disulfide reduction assay and shown in a histogram (*n* = 6 independent experiments). (c) Stat3 nuclear translocation in INS-1 cells was detected by immunofluorescence analysis and captured by fluorescence microscopy (100×) (*n* = 3 independent experiments). (d) Expression of *Trx2*, *Txnip*, *MafA*, *Pdx-1*, *Ins*, and *Stat3* was measured by qPCR in INS-1 cells after Irisin treatment. *β*-Actin was the internal standard (*n* = 3 independent experiments). (e) The secretory function of INS-1 cells is presented as the ratio of insulin secretion after 16.7 mM glucose treatment to insulin secretion after 3.3 mM glucose treatment. (f) Insulin secretion after 3.3 mM glucose or 16.7 mM glucose was measured by ELISA (*n* = 3 independent experiments). The data are shown as the mean ± SE; ∗*P* < 0.05 vs. Stat3 NC; ^#^*P* < 0.05 vs. Stat3 NC treated with Irisin. NC: nonsense control. (g) Protein expression of Trx2, Txnip, and Stat3 in INS-1 cells after Stat3 siRNA administration with or without PA and Irisin cotreatment was detected by immunoblotting and is shown in histograms (*n* = 3 independent experiments). (h) The secretory function of INS-1 cells is presented as the ratio of insulin secretion after 16.7 mM glucose treatment to that after 3.3 mM glucose treatment. (i) Insulin secretion after 3.3 mM glucose or 16.7 mM glucose stimulation was measured by ELISA (*n* = 6 independent experiments). The data are shown as mean ± SE; ∗*P* < 0.05 vs. the BSA+Stat3 NC group; ^#^*P* < 0.05 vs. the Stat3 siR+BSA group; ^&^*P* < 0.05 vs. the Stat3 siR+PA group. NC: nonsense control.

To confirm that Irisin-induced Trx2 expression resulted from Stat3 translocation, Stat3 inhibition induced by Stat3 siRNAs resulted in a blockage of Irisin-induced Stat3 expression (Figure 4(a)) and nuclear translocation (Figure 4(b)). Correspondingly, the increased intracellular Trx2 expression and activity caused by Irisin treatment were decreased by the inhibitory Stat3 siRNA effects (Figure 4(c)). Moreover, we found that Stat3 inhibition hindered the gene expression of *MafA* and *Ins*, which had been increased by Irisin treatment (Figure 4(d)), and enhanced insulin secretion induced by Irisin was also impeded by Stat3 inhibition (Figures 4(e) and 4(f)). These findings indicated that Trx2 expression was activated by Irisin through Stat3 nuclear translocation.

As shown in Figure 4(g), Irisin partially prevented Trx2 inactivation induced by PA treatment by decreasing Txnip expression, although Stat3 expression was inhibited by Stat3 siRNAs, confirming that Trx2 was activated by Irisin partially through Txnip inhibition. More importantly, Stat3 inhibition reversed the protective effect of Irisin on insulin secretion dysfunction induced by PA treatment, as shown in Figures 4(h) and 4(i).

### 3.5. In Vivo, Irisin Administration Preserves Pancreatic Beta-Cell Function and Enhances Glucose Tolerance in HFD-Fed Mice

**Figure 5:**
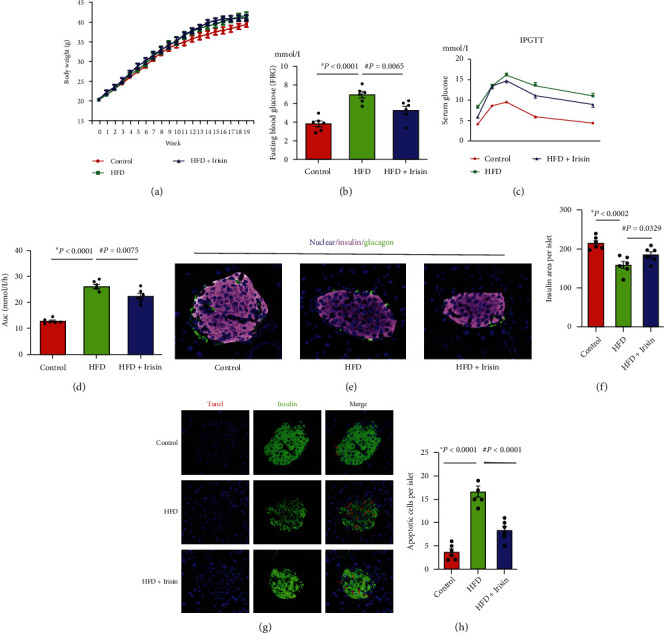
In vivo Irisin administration preserved pancreatic beta-cell function and enhanced glucose tolerance in HFD-fed mice. (a) Body weight of HFD-fed mice after Irisin treatment. (b) Three days after IPGTTs, the mice were euthanized after fasting overnight, and blood samples were collected to measure fasting blood glucose levels. Fasting glucose levels in the mice of different groups are shown in a histogram. (c) Intraperitoneal glucose tolerance test (IPGTT); (d) AUC of the IPGTT data shown in histogram. (e) Insulin content in pancreatic beta-cells as determined by immunofluorescence analysis. (f) Insulin content per islet was analyzed by ImageJ software and is shown in a histogram. (g) TUNEL analysis was performed to detect cell apoptosis of pancreatic beta-cells. (h) Apoptotic cells per islet analyzed by ImageJ software and shown in a histogram. The data shown as mean ± SE; ∗*P* < 0.05 vs. the control group; ^#^*P* < 0.05 vs. the HFD group. *n* = 6 mice in each group.

Intraperitoneal injection of Irisin in HFD-fed mice for 28 days resulted in no significant increase in body weight compared with a HFD-fed-only group mice (Figure 5(a)). However, Irisin administration led to a significant decrease in fasting glucose levels (Figure 5(b)) and recovery of glucose tolerance that had been disrupted by HFD treatment (Figures 5(c) and 5(d)). Compared with that in HFD-fed mice, the insulin content was increased in the pancreatic islets of mice injected with Irisin (Figure 5(e)). Moreover, Irisin administration markedly reduced beta-cell apoptosis within pancreatic islets, as shown in Figure 5(f).

**Figure 6:**
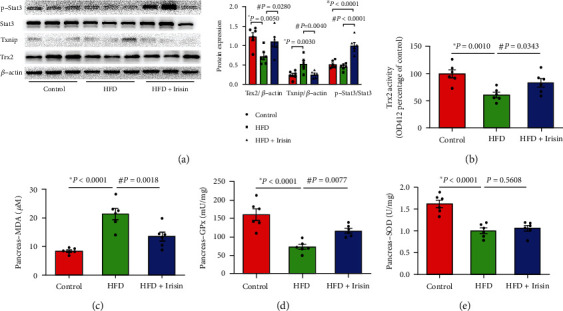
In vivo Irisin administration prevented HFD-induced Trx2 activation and oxidative stress. (a) The expression of Trx2, Txnip, p-Stat3, and Stat3 was detected by immunoblotting. *β*-Actin was an internal standard. (b) Trx2 activity in the pancreas was measured by insulin disulfide reduction assay [16]. (c) Pancreatic MDA content analysis in different groups. (d) Analysis of pancreas GPx activity in different groups. (e) Analysis of pancreas SOD activity in different groups. The data are shown as mean ± SE, ^∗^*P* < 0.05 vs. the control group; ^#^*P* < 0.05 vs. the HFD group. *n* = 6 mice in each group.

As shown in Figures 6(a) and 6(b), inhibition of Trx2 activity and increased expression of Txnip were detected in HFD-fed mice but were partially ameliorated after Irisin treatment. Irisin injection also increased the phosphorylation of Stat3 in HFD-fed mice. Correspondingly, Irisin treatment decreased the level of MDA and increased GPx activity compared to those in the HFD group (Figures 6(c) and 6(d)). However, Irisin treatment did not restore the SOD activity inhibited by HFD treatment (Figure 6(e)).

## 4. Discussion

The major findings in this study demonstrated that Irisin conferred protection against lipotoxicity-induced beta-cell dysfunction by inhibiting cell apoptosis and promoting insulin production and secretion. This effect might result from the activation of Trx2, which blocked oxidative stress. We proved that Txnip inhibition and Stat3 nuclear translocation were involved in Irisin-induced Trx2 activation in beta-cells, which is a new finding that explains the underlying mechanism of Irisin in pancreatic beta-cell protection.

Irisin, a newly discovered myokine, has been proven to promote the conversion of white fat to brown fat and regulate energy metabolism to attenuate obesity and reduce glucose intolerance induced by a high-fat diet [7]. A recent study discovered that recombinant Irisin prevented saturated fatty acid-induced beta-cell apoptosis of human and murine islets and released the inhibition of glucose-stimulated insulin secretion caused by PA treatment [10], indicating that Irisin is a potential beta-cell secretagogue and survival factor. In addition, Liu et al. found that Irisin protected beta-cells from high-glucose-induced apoptosis [11]. Therefore, to confirm the protective effects of Irisin, we conducted our study and found that recombinant Irisin similarly promoted INS-1 cell proliferation and inhibited PA-induced cell apoptosis, and this cytoprotective effect was partially reversed by an anti-Irisin-neutralizing antibody, indicating that INS-1 cells were protected from PA-induced cell injury after Irisin treatment.

The mechanism of Irisin-induced beta-cell survival in hyperglycemia or hyperlipidemia has not been clearly described. Irisin has been shown to ameliorate the disrupted oxidative homeostasis in endothelial cells caused by glucolipotoxicity and to enhance endothelial function through activation of the PKC-*β*/NADPH oxidase and NF-*κ*B/iNOS pathways [8]. Increased intracellular ROS after PA administration in INS-1 cells was found in our study, indicating that disruption of redox homeostasis may be involved in the effects of PA. Notably, PA and Irisin cotreatment significantly hindered PA-induced oxidative stress by decreasing ROS and MDA levels in INS-1 cells. Hence, Irisin showed antioxidant potential in pancreatic beta-cells, which was confirmed by Irisin NA cotreatment, which inhibited the antioxidative ability of Irisin. Nevertheless, we have no clue to explain the underlying mechanism of the antioxidative effect of Irisin on beta-cells.

Our previous study revealed that the mitochondrial antioxidant enzyme Trx2 might be critical for HG-induced oxidative stress and cell dysfunction [16], indicating that Trx2 plays antioxidant and antiapoptotic roles in beta-cell injury. In this study, we explored an increase in mitochondrial ROS in PA-treated INS-1 cells compared to a BSA group, but PA and Irisin cotreatment significantly decreased mitochondrial ROS levels compared to the levels in the PA group, implying that Trx2 might be related to Irisin-induced inhibition of oxidative stress. Therefore, INS-1 cells were treated with Irisin for different time durations, and we found that the expression of Trx2 increased in a time-dependent manner. PA inhibited Trx2 expression and activity, but Irisin and PA cotreatment prevented this inhibition of Trx2. Irisin NA cotreatment blocked the preventive effect of Irisin on Trx2 activation, confirming our hypothesis. We also found that PA treatment increased the level of Txnip in INS-1 cells and that Irisin significantly inhibited the expression of Txnip, which might be critical for Trx2 activation.

The mechanism by which Irisin regulates Trx2 expression in beta-cells has not been fully elucidated. Signal transducer and activator of transcription 3 (Stat3) belongs to the superfamily of signal transducer and activator of transcription proteins and is an important intracellular protective regulator. Recently, Stat3 has been found to affect mitochondrial ROS production and participate in the regulation of mitochondrial function [17, 18]. In our study, we found that Irisin treatment dramatically increased Stat3 expression and phosphorylation. Moreover, Irisin triggered Stat3 translocation from the cytoplasm to the nucleus, accompanied by an increase in Trx2 expression, but Irisin NA impeded the action of Irisin on Stat3 nuclear translocation. Stat3 activation might be critical for the increase in Trx2 expression caused by Irisin treatment because blockade of Stat3 significantly inhibited Stat3 nuclear translocation and prevented Irisin-induced Trx2 expression. PA treatment also inhibited Trx2 expression, probably due to the increased expression of Txnip but not Stat3 inactivation, as no significant inhibition of Stat3 translocation was detected after PA treatment. Irisin prevented PA-induced inactivation of Trx2 by decreasing Txnip expression independent of Stat3 activation, confirming that Trx2 was activated by Irisin partially through Txnip inhibition.

We further explored that HFD treatment significantly increased fasting blood glucose (FBG) and impaired glucose tolerance in vivo. These results were probably attributed to the increased apoptosis of pancreatic beta-cells and decreased insulin formation caused by HFD consumption, compared to those in the normal diet treatment, which confirmed lipotoxicity in beta-cells. However, Irisin injection partially reversed the cytotoxic effects of HFD treatment by attenuating beta-cell apoptosis and increasing insulin content, as well as lowering the level of blood glucose. Similarly, we detected decreased activation of Trx2 and increased expression of Txnip in the HFD treatment group, but Irisin administration reversed this outcome. The decreased level of MDA and increased activity of GPx further proved the antioxidative effect of Irisin on beta-cells. We found that Irisin increased Stat3 phosphorylation in pancreatic tissue, further indicating that Stat3 might contribute to Trx2 regulation.

However, we have no concrete evidence to explain the mechanisms of the Irisin effect on insulin secretion, although we detected increased expression of insulin after Irisin treatment. As reported previously, the increase in Txnip may induce miR-204 production by decreasing the activity of Stat3, a transcription factor involved in miR-204 regulation, and further reduce the expression of *MafA*, a downstream target of miR-204, to control insulin production in beta-cells; notably, miR-204 has been shown to be involved in beta-cell function [25]. In our study, we identified a significant decrease in Txnip expression and an increase in Stat3 nuclear translocation after Irisin treatment. Stat3 inhibition by Stat3 siRNAs also attenuated the increased expression of *Ins* and *MafA* induced by Irisin and decreased insulin secretion after high-glucose stimulation compared to the effects in the Irisin treatment. Stat3 inhibition reversed the protective effect of Irisin on insulin secretion dysfunction induced by PA. These findings indicated that the novel Txnip/Stat3/MafA/insulin pathway may be involved in Irisin-induced beta-cell protection. We did not find the receptor of Irisin in beta-cells in our study, although *α*V/*β*5 integrin had been previously identified as a receptor of Irisin in bone and adipose tissues [26], and activation of integrin *α*V*β*5-Akt signaling has been found to be critical for the cardioprotective effects of Irisin, which are mediated through its attenuation of oxidative/nitrosative stress [27]. Further studies will focus on identifying an Irisin receptor in beta-cells.

There are some limitations to this study. We chose a HFD model but not a diabetic model to prove the protective effects of Irisin on lipotoxicity in beta-cells in vivo. In vitro, the mechanisms of Irisin on Trx2 activation through Txnip inhibition and Stat3 activation need to be tested in primary islets.

## 5. Conclusions

In summary, these findings regarding the molecular mechanisms of Irisin in beta-cell apoptosis and dysfunction may revolutionize our comprehensive understanding of the protective effects of Irisin on pancreatic beta-cells and may provide a new therapeutic approach for protecting against pancreatic beta-cell injury. Further studies will be conducted to examine the underlying mechanisms of Irisin-induced Stat3 activation on Trx2 regulation in beta-cells.

## Figures and Tables

**Table 1:** Sequences of primers.

Primer name	Sequences (5′-3′)
*β*-Actin forward	GATTACTGCCCTGGCTCCTAG
*β*-Actin reverse	GAAAGGGTGTAAAACGCAGCTC
Trx1 forward	GGTGAAGCTGATCGAGAGCAAGG
Trx1 reverse	GCAGAGAAGTCCACTACCACAAGC
Trx2 forward	GGTAGCCAAACAGCACGGGAAG
Trx2 reverse	CAGCAGACACCTCGTACTCAATGG
Txnip forward	GCCAGACCAAAGTGCTCACTCAG
Txnip reverse	GAGACTCTTGCCACGCCATGATG
Stat3 forward	AGGGCTTCTCGTTCTGGGTCTG
Stat3 reverse	CTCCCGCTCCTTGCTGATGAAAC
Pdx-1 forward	GGTGCCAGAGTTCAGTGCTAATCC
Pdx-1 reverse	GACTTCCCTGTTCCAGCGTTCC
MafA forward	CCACCACCACGGAGGCTCTG
MafA reserve	TCCCGCACTGACATGGACACC
Ins forward	TGGTTTCTTCTACACACCCAAG
Ins reserve	CCACAATGCCACGCTTCT

## Data Availability

The data used to support the findings of this study are available from the corresponding author upon request.
